# Enhancing DNT Detection by a Bacterial Bioreporter: Directed Evolution of the Transcriptional Activator YhaJ

**DOI:** 10.3389/fbioe.2022.821835

**Published:** 2022-02-14

**Authors:** Tal Elad, Benjamin Shemer, Shilat Simanowitz, Yossef Kabessa, Yosef Mizrachi, Azriel Gold, Etai Shpigel, Aharon J. Agranat, Shimshon Belkin

**Affiliations:** ^1^ Department of Plant and Environmental Sciences, Institute of Life Sciences, The Hebrew University of Jerusalem, Jerusalem, Israel; ^2^ Department of Applied Physics and the Brojde Center for Innovative Engineering and Computer Science, The Hebrew University of Jerusalem, Jerusalem, Israel

**Keywords:** bioreporter, biosensor, directed evolution, explosives, landmines, LysR family, 2,4-dinitrotoluene

## Abstract

Detection of buried landmines is a dangerous and complicated task that consumes large financial resources and poses significant risks to the personnel involved. A potential alternative to conventional detection methodologies is the use of microbial bioreporters, capable of emitting an optical signal upon exposure to explosives, thus revealing to a remote detector the location of buried explosive devices. We have previously reported the design, construction, and optimization of an *Escherichia coli*-based bioreporter for the detection of 2,4,6-trinitrotoluene (TNT) and its accompanying impurity 2,4-dinitrotoluene (DNT). Here we describe the further enhancement of this bioreporter by the directed evolution of YhaJ, the transcriptional activator of the *yqjF* gene promoter, the sensing element of the bioreporter’s molecular circuit. This process resulted in a 37-fold reduction of the detection threshold, as well as significant enhancements to signal intensity and response time, rendering this sensor strain more suitable for detecting the minute concentrations of DNT in the soil above buried landmines. The capability of this enhanced bioreporter to detect DNT buried in sand is demonstrated.

## Introduction

The high risks involved in most prevalent methodologies for buried landmine detection, which require the on-site presence of personnel, have created an acute need for a standoff detection technology. To date, no commercially available technology meets this need.

The primary explosive found in most landmines is 2,4,6-trinitrotoluene (TNT), which is often accompanied by a 2,4-dinitrotoluene (DNT) manufacturing impurity. Vapors of both TNT and DNT have been reported to exist in soils above buried landmines (Sylvia et al., 2000; Jenkins et al., 2001). These vapors migrate to the surface through plastic components or from cracks in the casing, and their identification has served as a basis for diverse approaches for landmine bio-detection (Habib, 2007; Smith et al., 2008). The relatively volatile and stable DNT is considered an excellent signature chemical for the presence of TNT-based explosive devices (Jenkins et al., 2001).

Microbial bioreporters are genetically engineered microbial strains, “tailored” to report the presence of chemical targets (Van der Meer and Belkin, 2010; Elad and Belkin, 2016). The recombinant constructs harbor a fusion of a gene promoter, involved in the cellular response to the target, and a reporter gene(s), the expression of which yields a quantifiable output. The most commonly used reporter elements are the *lacZ*, *gfp*, or *lux* genes, yielding chromatic, fluorescent, or bioluminescent signals.

The use of microbial bioreporters for sensing TNT or DNT vapors in soil, indicating the location of buried explosive devices, has been proposed already in 1999. A number of bacterial bioreporters for the detection of traces of explosives have since been described (Burlage et al., 1999; Altamirano et al., 2004; Radhika et al., 2007; Garmendia et al., 2008; Kim et al., 2008; de las Heras and de Lorenzo, 2011; Davidson et al., 2012; Lönneborg et al., 2012; Yagur-Kroll et al., 2014; Yagur-Kroll et al., 2015; Tan et al., 2015; Belkin et al., 2017; see Shemer et al. (2015, 2017) for reviews). Prominent among these reports is a description of *E. coli*-based DNT/TNT bioreporters harboring a genetic fusion between *E. coli*’s endogenous *yqjF* gene promoter to either the green fluorescent protein gene *GFPmut2* or to *Photorhabdus luminescens* bioluminescence *luxCDABE* genes (Yagur-Kroll et al., 2014, 2015). While the fluorescent variant has been instrumental in demonstrating the standoff detection of real antipersonnel landmines (Belkin et al., 2017), recent efforts have concentrated on molecularly enhancing the performance of the bioluminescent variants (Shemer et al., 2020; 2021; Shpigel et al., 2021), in parallel to unravelling the DNT degradation pathway (Shemer et al., 2018) and the *yqjF* regulatory mechanism in *E. coli* (Palevsky et al., 2016). The latter study has pointed at YhaJ, a member of the LysR type family of transcriptional regulators, as a positive regulator of *yqjF* activation, linked to aromatic compounds degradation.

A close look at the published characteristics of previously described bacterial explosives’ sensor strains reveals that their performance may need to be significantly enhanced before their use can be considered for landmine detection under actual field conditions (Shemer et al., 2015, 2017). For example, equilibrium headspace concentrations of DNT and TNT vapors above TNT based landmines can be as low as 0.28 pg/ml and 0.077 pg/ml, respectively (Jenkins et al., 2001). Not all reported detection thresholds are sufficiently low to detect such low concentrations.

The aim of the present study was to improve the performance of the *yqjF*-based bacterial bioreporter by manipulating its regulatory protein YhaJ. This was achieved by a directed evolution of both the *yhaJ* gene and its promoter, carried out by three sequential random mutagenesis cycles. The selected final construct displayed superior DNT detection capabilities both in aqueous media and over a sand target.

## Materials and Methods

### Chemicals

Analytical grade DNT was purchased from Sigma-Aldrich. An ethanol stock solution (27 g/L) was kept at 4°C and diluted according to need. Sodium alginate (CAS 9005-38-3) and polyacrylic acid (PAA, CAS 9003-01-4, M_w_ ∼250,000, 35 wt% in H_2_O) were purchased from Sigma-Aldrich (Israel).

### Plasmids

**FIGURE 1:**
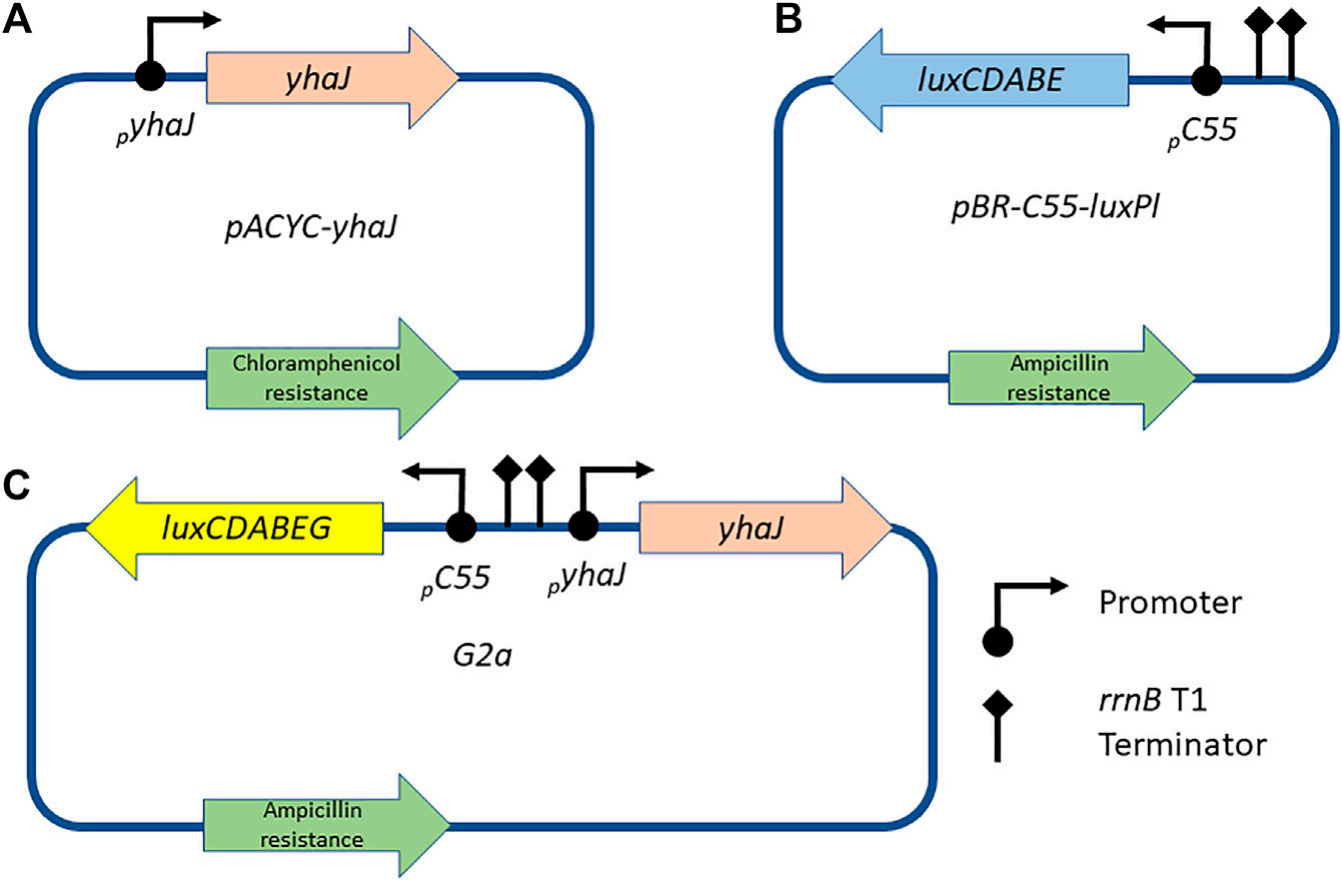
Schematic designs of the plasmids used in this study. The *yhaJ* gene, fused to its native promoter, is mounted on a pACYC platform **(A)**; in the two-plasmid system, this plasmid is co-transformed with plasmid pBR-C55-luxPl **(B)**, which carries a fusion between the C55 promoter and the *luxCDABE* genes of *P. luminescens*. Alternatively, in the one-plasmid system, all components are mounted on a single pBR2TTS backbone, carrying a *P. leiognathi luxCDABEG* reporter gene cassette [plasmid G2a, **(C)**].

To monitor the effects of sequence manipulations of the *yhaJ* gene and promoter, two separate plasmids were initially employed in the same *E. coli* host (referred to hereafter as the two-plasmid system). Plasmid pACYC-*yhaJ* (Figure 1A), a derivative of plasmid pACYC184 (Chang and Cohen, 1978), harbored the complete *yhaJ* gene, driven by its own original promoter. The promoter region and coding sequence of *yhaJ* were obtained by PCR amplification from the *E. coli* genome, introducing SphI and SalI restriction sites with primers yhaJ-SphI and yhaJ-SalI (Supplementary Table S1). The PCR products were gel-purified, digested with SphI and SalI, and ligated into the same restriction sites in pACYC184. The second plasmid, pBR-C55-luxPl (Figure 1B), contained a fusion between the *Photorhabdus luminescens luxCDABE* genes and C55, a mutated version of the *yqjF* promoter (Shemer et al., 2020).

A single-plasmid system (plasmid G2a, Figure 1C) was obtained by cloning a mutated version (G2) of the *yhaJ* gene into plasmid pBR-C55-luxPleio (Shemer et al., 2021), in which the C55 promoter is fused to the *luxCDABEG* genes of *Photobacterium leiognathi*. The G2 variant was PCR-amplified using primers 115_F and 116_R (Supplementary Table S1); NEBuilder HiFi DNA Assembly kit (New England Biolabs, United States) was used to assemble the two fragments.

The *E. coli* host strains and the plasmids employed or constructed in the course of the present study are listed Table 1. Full DNA sequences of the plasmids used in the course of this study are available as supplementary FASTA files (pBR-C55-luxPl.fasta, pBR-C55-luxPleio.fasta, pACYC-yhaJ.fasta, G2a.fasta).

**TABLE 1:** Strains and plasmids used in the course of this study.

Host strain	Description	Reference
DH5*α*	Standard cloning strain
BW25113 Δ*yhaJ*	A member of the Keio collection, hosting a single gene deletion of the *yhaJ* gene	Baba et al. (2006)

**Plasmid**	**Description**	**Reference**
pACYC-*yhaJ* (also denoted G0)	A copy of the *yhaJ* gene and promoter, mounted on a pACYC184 (Chang and Cohen, 1978) backbone	This work
G1	1st generation of *yhaJ* random mutagenesis	This work
G2	2nd generation of *yhaJ* random mutagenesis	This work
G2a	2nd generation of *yhaJ* random mutagenesis, mounted on a pBR-C55-luxPleio backbone	This work
G3a	3rd generation of *yhaJ* random mutagenesis	This work
G3b	3rd generation of *yhaJ* random mutagenesis	This work
G11T	G2a plasmid supplemented with a G11→T mutation	This work
A51G	G2a plasmid supplemented with a A51→G mutation	This work
T133A	G2a plasmid supplemented with a T133→A mutation	This work
G11T-A51G	G2a plasmid supplemented with G11→T and A51→G mutations	This work
G11T-T133A	G2a plasmid supplemented with G11→T and T133→A mutations	This work
A51G-T133A	G2a plasmid supplemented with A51→G and T133→A mutations	This work
pBR-C55-luxPl	A sensor-reporter plasmid, carrying a fusion between the C55 promoter and the *luxCDABE* genes of *P. luminescens*	Shemer et al. (2020)
pBR-C55-luxPleio	A sensor-reporter plasmid, carrying a fusion between the C55 promoter and the *luxCDABE* genes of *P. leiognathi*	Shemer et al. (2021)

### Random Mutagenesis

The first two rounds of *yhaJ* mutagenesis were performed using the two-plasmid system. Error-prone PCR was conducted according to Kagiya and colleagues (Kagiya et al., 2005) with slight modifications. The reaction contained 1 mM dCTPs, 1 mM dTTPs, 0.2 mM dATPs, 0.2 mM dGTPs, 2.5 mM MgCl_2_, 1 mM MnCl_2_, 5 U/ml Taq DNA polymerase (Fermentas, #EP0402), and 50 pmol/ml of primers yhaJ-sphI and yhaJ-SalI (Supplementary Table S1). The error-prone PCR mixture (50 μL) was divided into five individual 10 μL aliquots to enhance the diversity of the resulting mutations. Following the error-prone PCR procedure, the reaction mixtures were pooled, purified, digested with SphI and SalI restriction enzymes, and ligated into the same restriction sites in pACYC. The products of the ligation reaction were used to transform *E. coli* strain BW25113 *ΔyhaJ* (Baba et al., 2006), already transformed with pBR-C55-luxPl, to generate a *yhaJ* variant library. Following each round of error-prone PCR, the library was screened for improved variants as described below. A third round of the *yhaJ* promoter and gene mutagenesis was conducted with the G2a plasmid as template, using primers 117_yhaJ_RM and 118_yhaJ_RM (Table S1). For this purpose, the G2a plasmid was digested with sbfI and stuI restriction enzymes, and the amplified mutated segment was inserted via Gibson assembly technique (NEBuilder HiFi DNA Assembly kit, New England Biolabs, United States).

Site-directed mutagenesis of selected point mutations was performed by Gibson assembly using primers modified with the desired substitution, and verified by sequencing.

DNA sequences of the various *yhaJ* (promoter and gene) generations constructed in the course of this study are available as a FASTA supplementary file (yhaJ-G1-G2-G3a-G3b.fasta).

### Clone Library Screening and Storage

After each round of random mutagenesis, approximately 500 colonies were screened for improved variants as follows: the colonies were picked into 96-well microtiter plates, each well containing 150 μl of lysogeny broth (LB) supplemented with kanamycin (50 mg/L), ampicillin (100 mg/L), and chloramphenicol (30 mg/L). Also picked into the same set of 96-well microtiter plates were two colonies of the best performing variant of the previous generation. The 96-well microtiter plates were incubated overnight at 37°C with shaking (200 rpm), and 10-μl aliquots were then transferred into white 96-well microtiter plates with a transparent bottom, each well of which contained 90 μl of LB with or without DNT (2 mg/L) and 2% ethanol. The plates were incubated at 37°C and luminescence intensity (in the plate reader’s arbitrary relative light units, RLU) and optical density at 600 nm (OD_600_) were measured with a microplate reader (Infinite® 200 PRO, Tecan) at 45 min intervals for several hours. Variants that displayed a higher response ratio (light intensity in the presence of DNT divided by that in its absence) and a higher (or similar) signal intensity compared to the best performing variant of the previous generation were selected for further analysis. The remaining 130 μl of each overnight culture were augmented with 130 μl of 50% glycerol solution and stored at −80°C.

### Exposure of Cells to DNT, Liquid Medium

Stored bacteria were plated on LB-agar Petri dishes supplemented with ampicillin (100 μg/ml), chloramphenicol (30 mg/L), and kanamycin (50 mg/L). Fresh colonies were grown overnight in LB with the same antibiotic composition at 37°C with shaking (200 rpm). Bacteria were diluted 100-fold in fresh LB and regrown under the same conditions to the mid-exponential growth phase (OD_600_ ≈ 0.3). Aliquots (50 μl) were pipetted into a white 96-well microplate with a transparent bottom (Greiner Bio-One) containing a twofold dilution series of DNT (double-distilled water, 4% ethanol) in the same volume. Light intensity (RLU) and absorbance (OD_600_) were measured with a microplate reader (Infinite^®^ 200 PRO, Tecan) at 12.5 min intervals at ambient temperature. All experiments were repeated at least three times.

### Performance Evaluation

Bacterial bioreporter performance was evaluated using four main parameters: signal intensity, response ratio, detection threshold, and response time. These parameters were defined as follows: *Signal intensity*, the amount of light emitted by the culture in relative light units (RLU), divided by the culture’s optical density (OD_600_); *Response ratio*, signal intensity of the induced sample divided by that of the non-induced reference; *Detection threshold*, the DNT concentration that would promote a response ratio of 2 as estimated by interpolation (EC_200_; Belkin et al., 1997); *Response time*, the time point at which a response ratio of two was first exceeded.

### Immobilization of Microbial Biosensors in Ca-Alginate Beads

Ca-alginate, supplemented by polyacrylic acid (PAA), was used to immobilize the bacteria for the detection of DNT buried in soil as previously described (Shemer et al., 2021). A 2.5% (w/v) Na-alginate solution was prepared by dissolving 30 g of Na-alginate in 950 ml of deionized H_2_O. Next, 50 ml of a 10% (w/v) PAA solution, previously neutralized to pH 7.0 with 10 M NaOH, were added and the solution was kept at room temperature overnight to ensure a clear and homogenous solution. The bacterial strains were incubated overnight (37°C, 200 rpm) in 50 ml LB supplemented with the appropriate antibiotics, and then diluted x1/50 in 50 ml LB supplemented with the same antibiotics. The culture was regrown under the same conditions to an OD_600_ of 0.8–1.0. The bacteria were then centrifuged (20 min, 6,000 rcf, 4°C, Sorval RC5C) and the pellet was weighed and resuspended in 5 ml 0.9% NaCl. An aliquot of resuspended bacteria was added to 500 ml of Na-alginate-PAA to reach a concentration of 0.1% (w/v). The bacterial suspension was dripped into 0.1 M CaCl_2_ using a Buchi B390 Encapsulator (Buchi Labortechnik AG, Switzerland) equipped with a 1,000 µm nozzle, at a pressure of 550 mbar. The formed beads were kept in CaCl_2_ for at least 30 min, then strained and washed with 0.9% NaCl. The washed beads [average diameter 3.97 (± 0.22 mm standard deviation), *n* = 17] were stored at 4°C until used. Cell concentration in the beads was enumerated by submerging 10 beads in 10 ml of a 2% (w/v) sodium citrate solution, until the beads were fully dissolved. Then, serial dilutions in 0.9% NaCl were plated on LB-agar plates containing the appropriate antibiotics and incubated overnight at 37°C. The average cell concentration was 1.82 × 10^5^ [± 1.9 × 10^4^ standard deviation] cells/bead.

### Exposure of Alginate-Immobilized Cells to DNT, Soil Surface

The bioluminescent response of the G2 and G2a bacterial bioreporters was measured on a soil surface using an imaging system comprised of a sensitive camera and an optical system for imaging the emittance of the bacteria onto the camera’s sensor array. The camera employed was a cooled scientific CMOS camera (PCO.edge5.5; PCO, Germany), characterized by a very low noise, a wide dynamic range, a rapid frame rate, and a high resolution. The optical system, designed for optimal imaging of the bacterial bioluminescent signal, was based on a high precision aspherical lens (Kowa, model LM12XC), which reduces distortion and produces high-definition images. The imaging system was isolated from both optical and electronic background noise in a special chamber, in which ambient light, temperature and humidity were strictly controlled. A detailed description of this controlled chamber can be found elsewhere (Agranat et al., 2021).

A monolayer of beads was homogenously spread on triplicate 35 mm diameter targets, containing soil with different amounts of buried DNT, as well as DNT-free control targets. The target array was imaged continuously with the temperature kept at 25°C and the humidity set to 55%. The targets were imaged with varying exposure times.

To extract quantitative data out of the imaged bioluminescent response, image processing tools were applied. We first defined a region of interest in the samples' locations by applying thresholds (Otsu, 1979), detecting edges (Gao et al., 2010), and detecting shapes (Ballard, 1981). The average luminescence value was then calculated for each sample across at least seven similar images in each batch and three similar targets. The bioluminescent response was measured in nW/cm^2^, according to a previously established calibration procedure (unpublished). Light emitted by a LED light source, with an emission spectrum and an intensity range similar to bacterial bioluminescence, was simultaneously measured by the CMOS camera-based imaging system and by a calibrated power meter (model StarBright, MKS Ltd.). The response of the direct imaging system was found to be linearly correlated to the optical intensity measured by the power meter in physical units (nW/cm^2^).

Further demonstration of the detection capabilities was also performed by spreading immobilized bacterial bioreporters (strain G3b) on a sand target containing DNT “hot spots”. These were prepared by placing DNT crystals (100, 300 and 1,000 mg) on separate Petri dishes, covering the explosives with sand (Sigma-Aldrich, 50-70 mesh, 40 g, 12 mm depth), and integrating the plates in a large container (20 × 28 x 5 cm) filled with Mediterranean beach sand. A thin layer (ca. 1-2 mm) of beach sand covered the targets, obtaining a uniform surface. The target was left at room temperature for 5 months prior to the experiment, to allow permeation of DNT vapors to the surface. The target was lightly sprinkled with LB medium prior to applying the beads. After placing the beads, the target was incubated in a dark chamber at room temperature and photographed every 15 min using a Sony s7a ii camera (4 s exposure, ISO 400), placed 1 m above the target, for a total of 9 h. To quantify the emitted luminescence, the integrated grey value across 10 randomly selected individual beads located above each “DNT hotspot” was quantified with ImageJ analysis software (Schindelin et al., 2012).

## Results

We set out to improve the performance of *yqjF*-based bacterial bioreporters for landmine detection by the directed evolution of YhaJ, a transcriptional regulator of *yqjF* activation (Palevsky et al., 2016). Using error-prone PCR, two *yhaJ*-targeted sequential mutagenesis rounds were performed, in which the *yhaJ* variants were incorporated into a plasmid (Figure 1A) that was subsequently introduced into a *ΔyhaJ* strain. The recipient cells were already transformed with a plasmid-borne fusion of a *yqjF* promoter [variant C55, previously modified for enhanced performance (Yagur-Kroll et al., 2014, 2015; Shemer et al., 2020)] to the *P. luminescens* bioluminescence *luxCDABE* gene cassette (Figure 1B). The clone libraries were screened, and clones that displayed a higher response ratio and a similar or higher signal intensity compared to the previous generation were further analyzed. The strain harboring the unmodified plasmids displayed in Figure 1A and Figure 1B is referred to as G0, and the best performers of mutagenesis rounds 1 and 2 as G1 and G2, respectively. Transferring the G2 variant to a one-plasmid design has yielded clone G2a (Figure 1C), with which a third round of error-prone PCR was conducted. The two mutant clones selected from among the variants generated in the third mutagenesis cycle are referred to as G3a and G3b.

### Effect of the Directed Evolution Process on Signal Intensity, Response Ratio, Detection Sensitivity and Response Time

**FIGURE 2:**
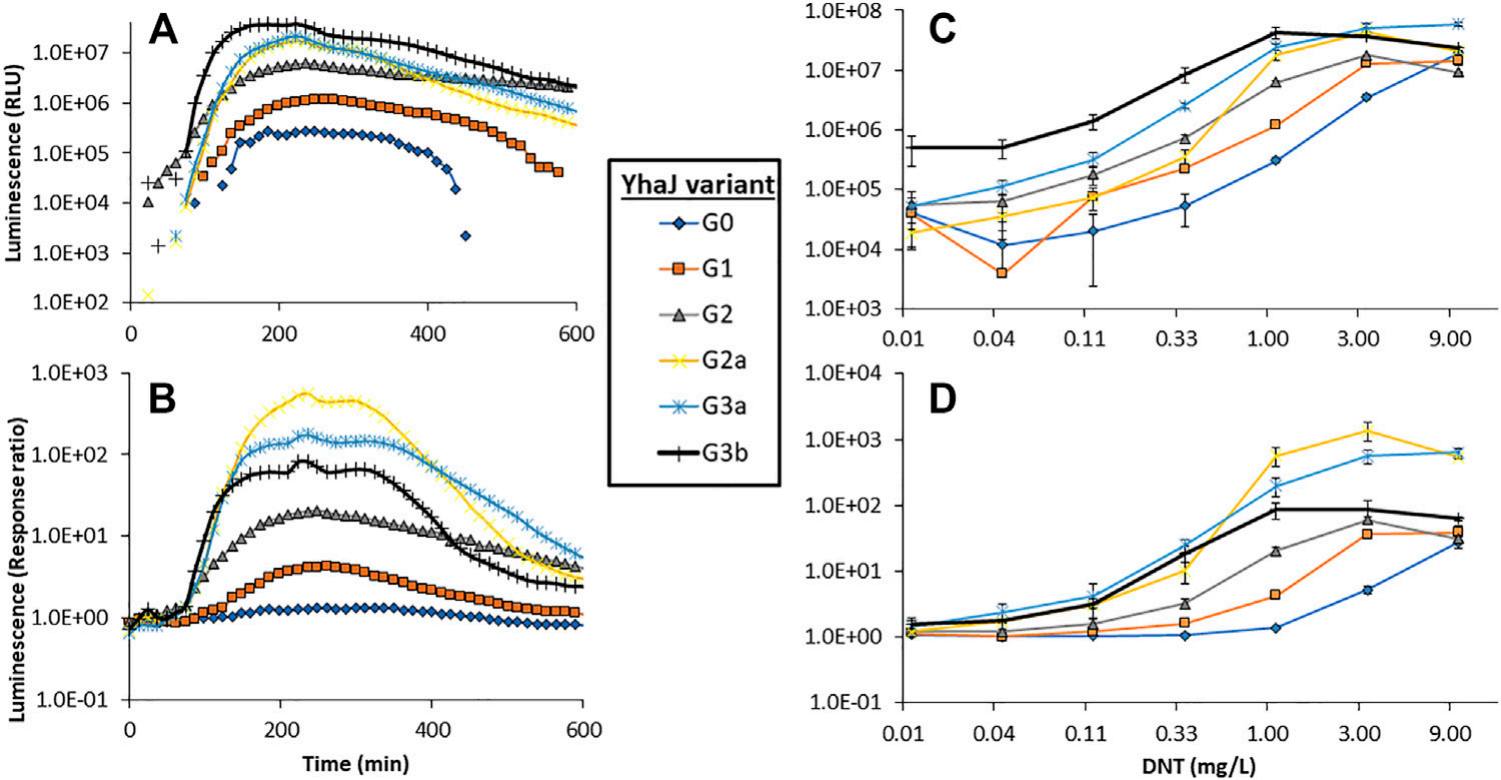
Dynamics of the luminescent response of the various *yhaJ* generations to a single DNT concentration (1.1 mg/L), displayed as signal intensity **(A)** and response ratio **(B)**. Panels **(C)** and **(D)** depict the maximal luminescence and maximal response ratios, respectively, over a 600 min exposure, as a function of DNT concentration for all *yhaJ* variants. Error bars represent the standard deviation across three repeats.

The response to DNT of selected clones from the different mutagenesis cycles is displayed in Figure 2. The signal intensity and response ratio dynamics of all variants were characterized by a lag phase followed by a dose-dependent increase, which peaked after 4–5 h. A clear increase in luminescence intensity as a function of the progress of the evolution process was observed (Figure 2A), a trend that was not fully replicated in the response ratios (Figure 2B), a parameter which is strongly affected not only by the increase in *yqjF* induction but also by changes in the control (uninduced) luminescence. The increases in the response ratio observed in clones G1 and G2 (Figures 2B,D) was mostly attributed to a significant decrease in the basal luminescence level with each generation. G2’s basal luminescence at time zero was similar to those of G0 and G1; however, it remained steady throughout the measurement and did not increase, as opposed to those of the two previous generations. Clones G3a and G3b, while superior to G2a in terms of signal intensity, suffered from a high background luminescence, which lowered their response ratios. The significant increase in light intensity from G2 to G2a, which harbors an identical *yhaJ* variant, is driven by the change of the *lux* reporter cassette from that of *P. luminescens* to *P. leiognathi* (Shemer et al., 2021).

**FIGURE 3:**
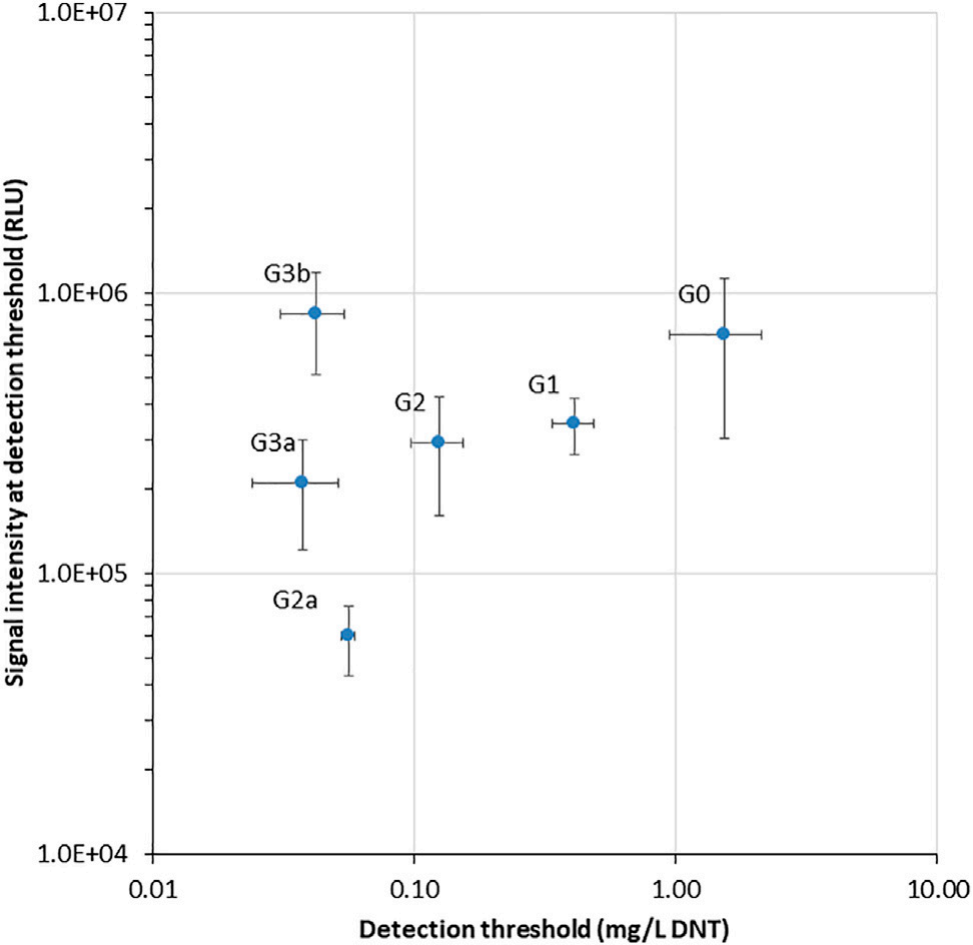
Luminescence intensity at the detection threshold for all *yhaJ* variants, as a function of the detection threshold (EC_200_: DNT concentration eliciting a response ratio of 2; Belkin et al., 1997). Error bars represent the standard deviation across three repeats.

Two additional performance parameters that were improved along the evolutionary progression were the detection threshold and the response time. In Figure 3, the light intensity at the detection threshold is plotted against the threshold DNT concentration. A clear progressive increase in sensitivity from G0 to G3 is observed; clone G3b appears to embody an optimal combination of a low detection threshold with high signal intensity. The detection threshold, calculated here as the DNT concentration promoting a response ratio of 2 (Belkin et al., 1997), decreased by 37-fold from 1.5 ± 0.6 mg/L DNT in the wild type to 0.04 ± 0.01 mg/L in the G3a and G3b variants (Figure 3 and Table 2). The response time, determined as the time point at which a response ratio of two was first exceeded, was reduced from 74 to 37 min and from 127 to 66 min for 10 and 3.3 mg/L DNT, respectively. At a DNT concentration of 0.12 mg/L, detection was not observed for generations G0-G2, but was apparent after 120-160 min in the G2a, G3a and G3b variants (Table 2).

**TABLE 2:** Response times, signal intensity and DNT detection sensitivity for the different *yhaJ* variants.

DNT concentration	10 mg/L		3.3 mg/L		0.12 mg/L		
*yhaJ* variant	Response time (min)^a^	Max signal intensity (RLU)^b^	Response time (min)^a^	Max signal intensity^b^ (RLU)	Response time (min)^a^	Max signal intensity^b^ (RLU)	Detection threshold (mg/L)^c^
G0	74	1.91E+07 ± 2.73E+06	127	3.54E+06 ± 3.79E+05	(−)#	2.03E+04 ± 1.79E+04	1.53 ± 0.587
G1	94	1.43E+07 ± 2.55E+06	111	1.31E+07 ± 1.34E+06	(−)	7.62E+04 ± 3.19E+04	0.41 ± 0.075
G2	49	9.18E+06 ± 2.56E+05	66	1.78E+07 ± 2.45E+05	(−)	1.82E+05 ± 6.36E+04	0.13 ± 0.028
G2a	53	2.15E+07 ± 1.84E+06	74	4.41E+07 ± 7.31E+06	156	7.36E+04 ± 6.87E+03	0.06 ± 0.003
G3a	58	5.84E+07 ± 4.10E+06	74	5.08E+07 ± 8.89E+06	123	3.23E+05 ± 8.94E+04	0.04 ± 0.014
G3b	37	2.32E+07 ± 1.62E+06	66	3.69E+07 ± 1.88E+06	152	1.40E+06 ± 3.88E+05	0.04 ± 0.012

a The time at which the response ratio first exceeded 2.

b In the course of a 600 min exposure; error values are calculated as standard deviation, resulting from at least three independent duplicate repeats.

c Minimal DNT concentration at which a response ratio of two was obtained.

#A response ratio higher than two was not obtained throughout the experiment.

### Mutations Introduced in the Course of the Directed Evolution Process

**FIGURE 4:**
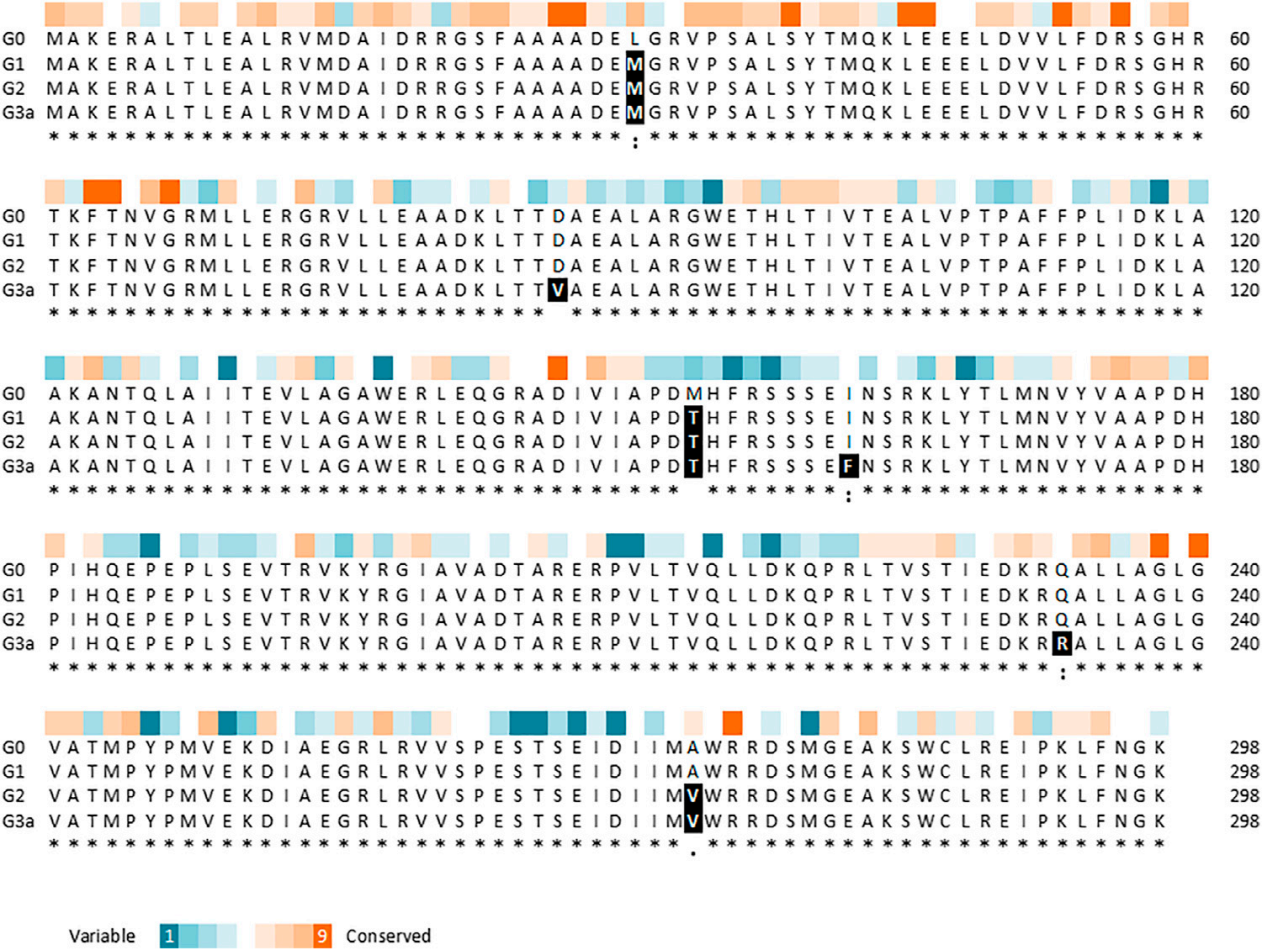
Multiple sequence alignment of YhaJ generations G0, G1, G2, and G3a, showing the amino acid replacements, as generated using the Clustal Omega tool (Sievers et al., 2011). The residue conservation scores of YhaJ, obtained with Consurf (Ashkenazy et al., 2016) based on 150 homologues from the UniRef database, are shown above the alignment.

Variant G2 carries three point mutations in *yhaJ*’s coding sequence (CDS), as revealed by sequencing: a cytosine-to-adenine conversion in position 91 of the CDS (g.91C > A), translating into a leucine-to-methionine substitution in position 31 of the amino acid (AA) sequence (p.L31M); a thymine-to-cytosine conversion in position 461 of the CDS (g.461T > C), translating into a methionine-to-threonine substitution in position 154 of the AA sequence (p.M154T); and a C-to-T conversion in position 812 of the CDS (g.821C > T), translating into an alanine to valine substitution in position 274 of the AA sequence (p.A274V; Figure 4). The first two point-mutations, p.L31M and p.M154T, were the result of the first round of random mutagenesis and are the ones carried by G1, while the third mutation, p.A274V, was introduced into *yhaJ*’s CDS in the second round.

To reduce potential instability due to plasmid imbalance, as well as to negate the need to transfect clones resulting from random mutagenesis with an additional plasmid before screening for activity, the third-generation variant (G2) of the *yhaJ* gene and promoter was mounted on the pBR-C55-*luxPleio* plasmid, yielding plasmid G2a. An additional round of mutagenesis of the *yhaJ* gene and promoter was conducted on this plasmid, and selected variants were transformed into a *yhaJ* deficient mutant. As noted above, the two mutant clones selected following this process were denoted G3a and G3b. The G3a variant has two mutations in its promoter sequence (C50 > A and A136 > G) as well as several mutations in its CDS. Three point-mutations (g.A260 > T, g.A484 > T, g.A698 > G) are reflected by AA substitutions (p.D87V, p. I162F, p. Q233 > R), while additional three codon substitutions (g.C54 > T, g.T600 > A, g.G762 > A) do not alter the AA sequence. In contrast, the G3b variant has only three point-mutations, all of which are positioned in the promoter region (G11 > T, A51 > G, T133 > A). The amino acid sequences of variants G0, G1, G2, and G3a are shown in Figure 4.

An estimation of the evolutionary conservation of the amino acid positions in YhaJ, based on the phylogenetic relations between homologous sequences, revealed, as expected, an agreement between the conservation level and the type of amino acid replacement. Amino acids in conserved positions were replaced with amino acids with similar properties, while other substitutions could be more radical (Figure 4). Querying the YhaJ AA sequence against the Protein Data Bank (PDB) (wwPDB consortium, 2019), we found a high degree of homology between YhaJ and CrgA, a LysR type transcription regulator (LTTR) from *Neisseria meningitidis*. This homology indicates that the mutations introduced here to YhaJ’s amino acid sequence span all of the protein’s domains, including the DNA binding domain (p.L31M), the linker helix (p.D87V), and the regulatory domains, in which substrate binding takes place (p.M154T, p. I162F, p. Q233R, and p. A274V) (Maddocks and Oyston 2008; Sainsbury et al., 2009).

**FIGURE 5:**
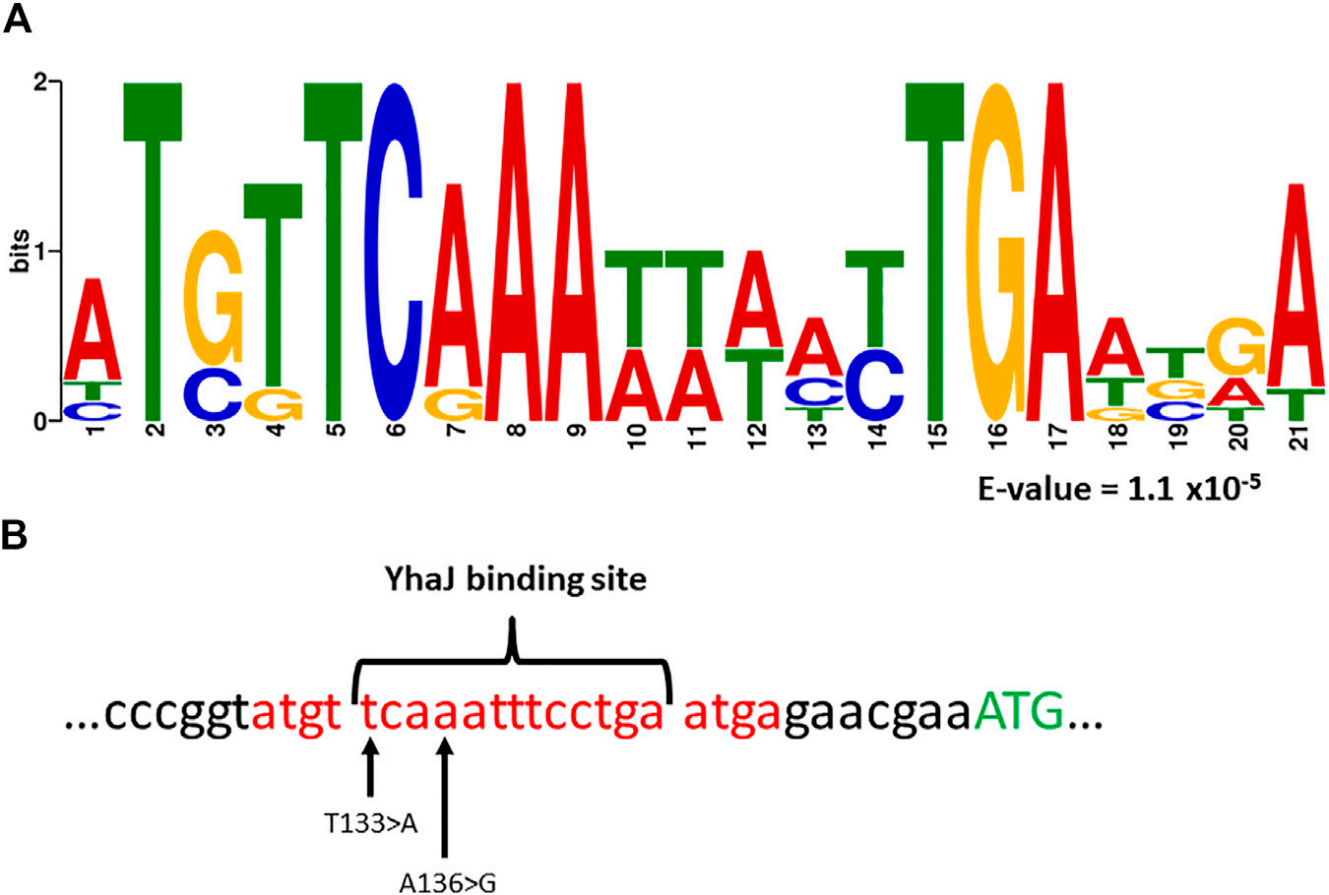
Panel **(A)**—a common motif found in the promoter regions of *yhaJ* and genes the activation of which is YhaJ-dependent (Palevsky et al., 2016; Henshke et al., 2021). Analysis was performed with the MEME suite algorithm (Bailey et al., 2006). The E-value represents the statistical significance of the motif as calculated by the MEME algorithm. Panel **(B)**—location of the sequence motif (marked red), as well as the first methionine of YhaJ (marked green), a known YhaJ binding site (Connolly et al., 2019, 2020), and the location of two point-mutations, T133 > A and A136 > G, found to significantly enhance the response of the bioreporter to DNT.

In previous studies, a common motif was found in YhaJ-regulated genes (Palevsky et al., 2016; Henshke et al., 2021). Interestingly, the MEME suite algorithm (Bailey et al., 2006), has revealed a significantly enriched (E-value of 1.1 × 10^−5^) sequence motif (Figure 5A). This motif is positioned on the (−) strand of the *yhaJ* promoter and contains a highly conserved binding site (Connolly et al., 2019; Connolly et al., 2020).

Variants G3a and G3b both host mutations located in this motif; A136 > G and T133 > A for G3a and G3b, respectively. Furthermore, in both cases the mutation is located at a highly conserved position of the YhaJ binding site (Figure 5B). This could hint at the importance of these two mutations in enhancing the activity of the bioreporter, as well as at the possible autoregulation of YhaJ. Autoregulation of LTTR’s has been shown to be a widespread genetic phenomenon (Maddocks and Oyston, 2008), but to the best of our knowledge has not been specifically shown in YhaJ.

**FIGURE 6:**
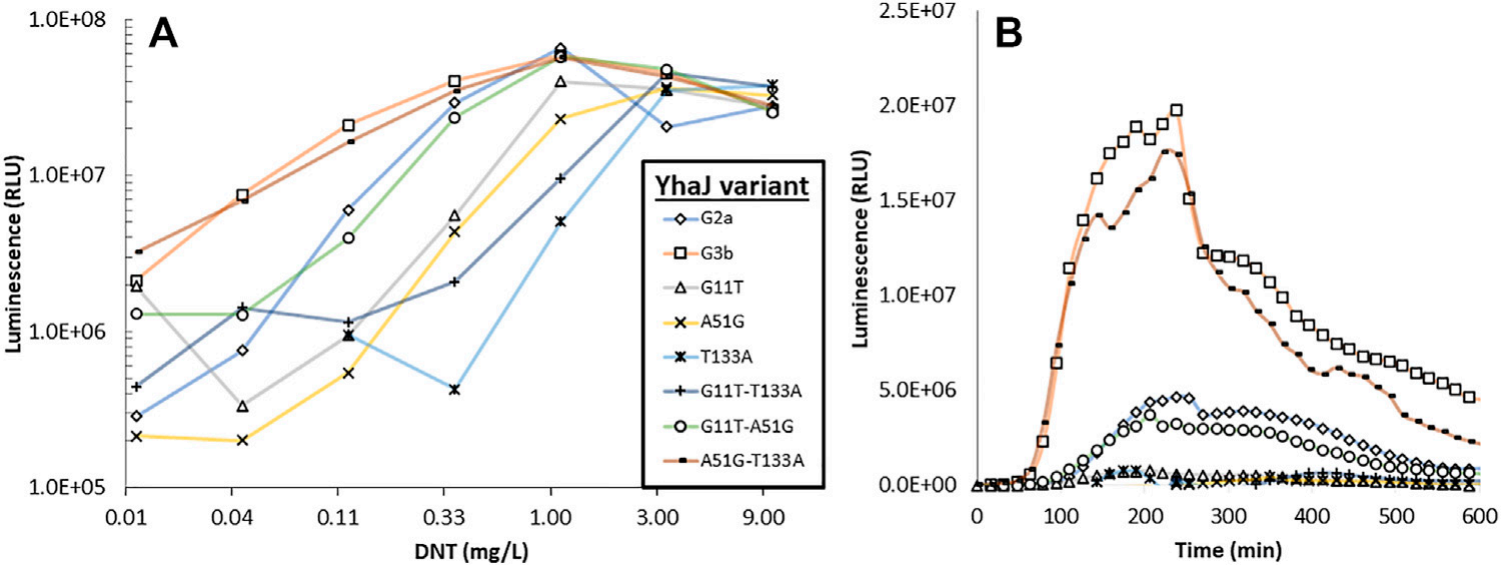
Luminescent response to DNT of strain G2a, to which the three mutations characterizing strain G3b have been introduced singly or in pairs. Panel **(A)**—maximal signal intensity observed for each strain in the presence of different DNT concentrations; panel **(B)**—response dynamics of all strains when spiked with 0.12 mg/L DNT.

To characterize the contribution of each mutation originating from the last round of mutagenesis to the effect demonstrated in the G3b strain, a set of G2a plasmids was constructed, supplemented with all the possible combinations of the identified single-point mutations. As depicted in Figure 6, no single mutation originating from the last round of mutagenesis is accountable for the increased response in G3b. However, when modifying G2a with both A51 > G and T133 > A mutations, the response of the resulting strain is practically similar to that of G3b.

### Rapid Imaging of the G2 Variant’s Luminescence: Comparing the Double-Plasmid (G2) and the Single-Plasmid (G2a) Clones

**FIGURE 7:**
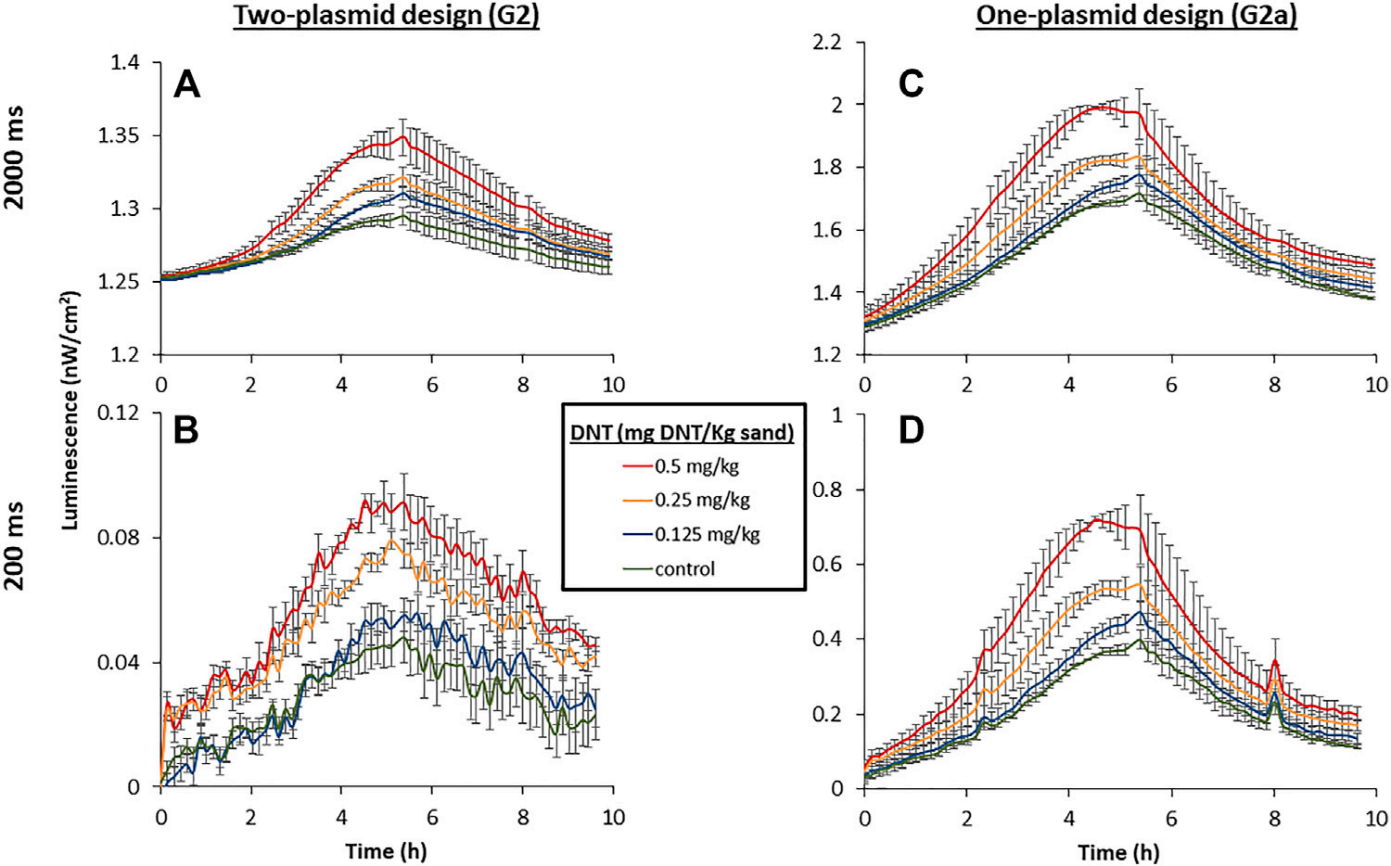
Luminescent response of the two-plasmid design [yhaJ (G2) + pBR-C55-Pleio; **(A,B)**] and the single-plasmid design [G2a; **(C,D)**] to DNT buried in sand. The targets were incubated at 25°C, 55% relative humidity, and imaged with a PCO. edge5.5 CMOS camera at constant intervals. A calibration process was employed to convert the measured luminescent signal to physical units (nW/cm^2^). Exposure times were 2000 ms **(A,C)** and 200 ms **(B,D)**.

The benefit of the increased light intensity displayed by the G2a variant is further underlined when performing rapid measurements, which may be essential in actual field applications. To demonstrate this, a sensitive cooled scientific CMOS camera, installed in a temperature- and humidity-controlled chamber, was employed to capture the signal emitted by immobilized bacteria placed on top of DNT-containing sand targets. The imaging was performed with two separate exposure times, 2000 and 200 ms. A two-plasmid design, harboring plasmids G2 and pBR-C55-luxPleio and containing the same relevant genetic parts, served for comparison. As demonstrated in Figure 7, the increased light emission of the G2a one-plasmid design enabled the detection of all tested DNT concentrations, even at the short exposure time (200 ms). In contrast, in the case of the two-plasmid system, it was not possible with this short exposure to differentiate the signal emitted in the presence of the low DNT concentration (0.125 mg/kg) from that of the background.

### Detection of Buried DNT by Variant G3b

**FIGURE 8:**
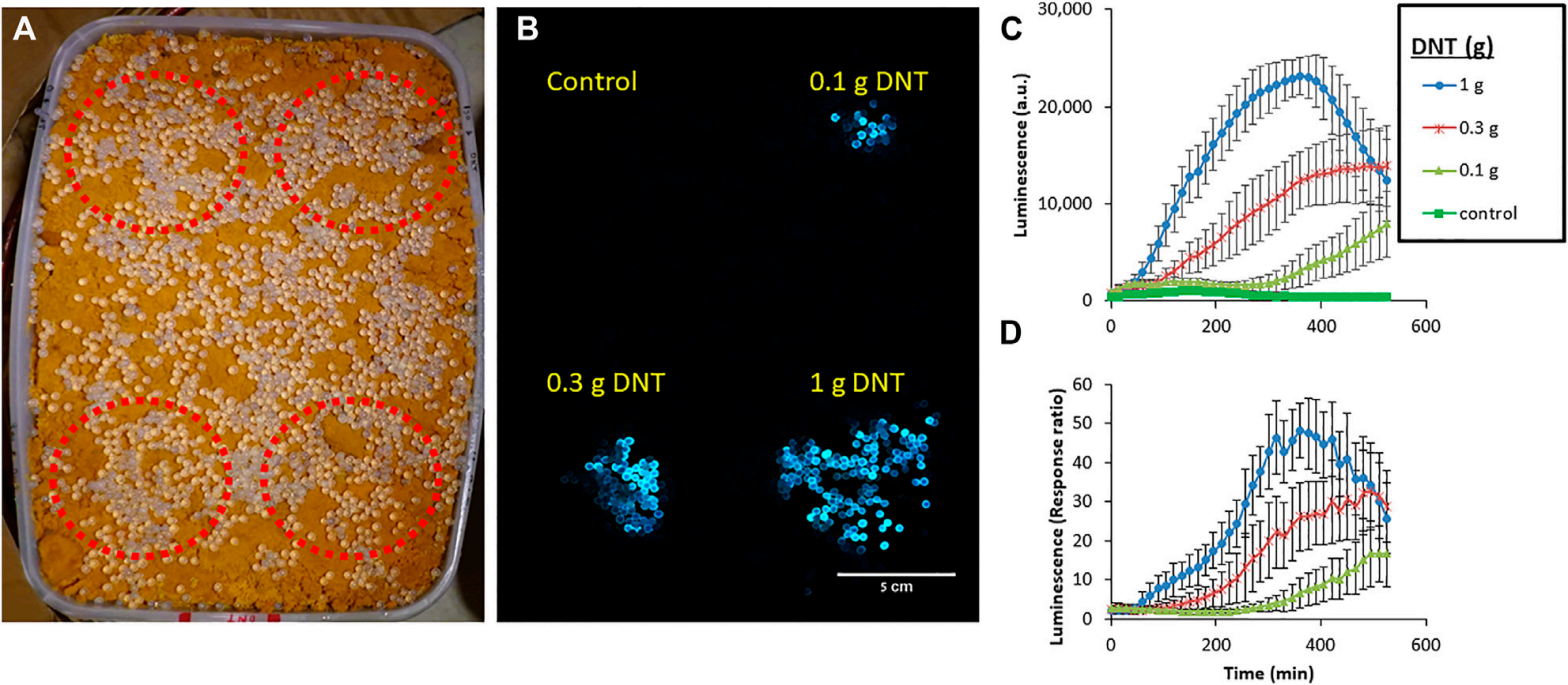
Detection of DNT buried in sand by the G3b variant. Immobilized bioreporters were spread across a target containing three DNT “hotspots” (marked in red), buried in a sand-filled container **(A)**, incubated at room temperature, and imaged every 15 min. **(B)**—an image taken after 9 h of incubation. **(C)**—Average signal intensity across 10 beads located above each hotspot, calculated by grey value integration. **(D)**—Response ratio measured above each hotspot. Imaging was performed with a Sony a7s ii camera (4 s exposure time, ISO 400) in a dark chamber, from a distance of ca. 1 m. Error bars represent the standard deviation based on luminescence measurement of nine random beads located above each target.

To demonstrate the detection capabilities of the most advanced *yhaJ* generation, G3b, the bacteria were immobilized in small (ca. 4 mm diameter) alginate beads and spread over a sand-filled container with three DNT “hotspots” (Figure 8A). After several hours, a clear luminescent response was visible, even with the camera’s modest imaging sensitivity parameters (Figure 8B). The images across 9 h of the experiment (Supplementary Video S1) were joined and analyzed by integrating the grey values across equal areas above the different hotspots. Interestingly, the luminescent signal across the entire target rose during the first 2 h of the experiment, then the background decreased, and the signal above the buried DNT could be clearly discerned (Figure 8C). The response time increased with decreasing DNT concentrations; a response ratio of two was observed after 105 min above 1 g of DNT, while 225 and 375 min were required to obtain the same response ratio for 0.3 and 0.1 g DNT, respectively (Figure 8D).

## Discussion

The microbial sensor strain at the core of the present study harbors a fusion of the *E. coli yqjF* gene promoter to bioluminescence reporter genes. In previous studies we have significantly enhanced the performance of this bioreporter by manipulating the sequence of the promoter region (Yagur-Kroll et al., 2015) and by introducing mutations into the host strain (Shemer et al., 2020), as well as by varying the origin of the reporter genes (Shemer et al., 2021). In the present study we have adopted a different approach: modifying YhaJ, the LysR-type transcriptional regulator of *yqjF* (Palevsky et al., 2016), by inserting into it potentially beneficial mutations by error-prone PCR. After three rounds of mutagenesis we have isolated a *yhaJ* variant (G3b) hosting point-mutations in both its promoter and its coding sequence, the activity of which was highly superior to that of the parent strain. Its maximal luminescent signal intensity was 69-fold higher (in the presence of 0.12 mg/L DNT), its DNT detection threshold 37-fold lower, and its response time (in the presence of 3.3 mg/L DNT) was reduced by ca. 50% (Table 2). Furthermore, we have demonstrated the capability of this enhanced luminescent bioreporter to detect DNT buried in soil with a relatively simple imaging device.

**FIGURE 9:**
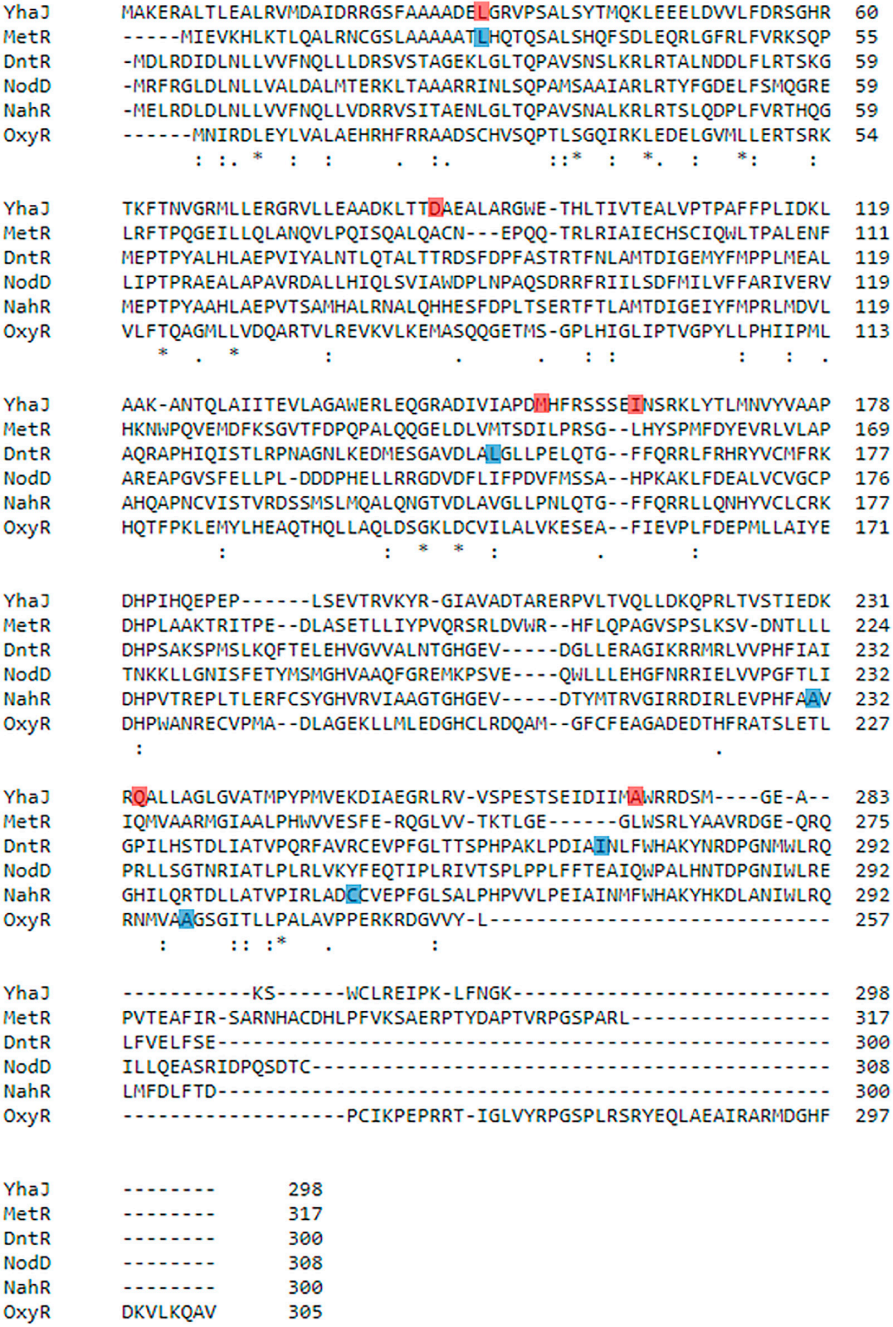
Multiple sequence alignment of YhaJ and selected LTTRs, as generated using the Clustal Omega tool (Sievers et al., 2011). YhaJ (WT): *Escherichia coli*, NCBI Reference Sequence: NP_417576.1; MetR: *E. coli*, UniProtKB Entry Identifier: P0A9F9; DntR: *Burkholderia* sp, UniProtKB Entry Identifier: Q7WT50; NodD: *Rhizobium meliloti*, UniProtKB Entry Identifier: P03031; NahR: *Pseudomonas putida*, UniProtKB Entry Identifier: P10183; OxyR: *E. coli*, UniProtKB Entry Identifier: P0ACQ4. Positions that were altered in this study are marked in red. Other positions mentioned in the main text are marked in blue.

Putting the CDS mutations in a wider context reveals additional details about their unique locations in YhaJ’s secondary and tertiary structures. The activity of a single point mutation clone carrying L31M was comparable to that of the wild type (data not shown), as was a substitution of serine for L26 in the LTTR MetR (Maxon et al., 1990). The M154T mutation by itself could also not account for the G0 to G1 improvement; only when combined, have these two mutations brought about the phenotype exhibited by G1 (data not shown). M154 and A274 are situated next to conserved or semi-conserved positions (I)150 and (I)271, respectively (Figure 4). In the LTTR DntR, which has been isolated from a *Burkholderia* species that is able to degrade 2,4-DNT, the amino acids in these positions (L151 and I273; Figure 9) were predicted to form direct hydrogen bonds with the carboxyl and hydroxyl groups of salicylate as an inducer (L151) and to stabilize its aromatic ring by hydrophobic interactions (I273; Smirnova et al., 2004). Therefore, M154 and A274 may directly interact with Yhaj’s ligand, providing the protein with its specificity. Similar to M154 and A274, Q233 is located in a small flanking region of a conserved domain, which contains a part of the inducer-binding crevice predicted for the LTTR NodD (Figure 4 and Figure 9; Schell 1993; Györgypal and Kondorosi 1991). Mutations in this region alter the responses of LTTRs to their inducers, highlighting its importance to the multiple functions of this protein family. For example, substitution mutations at positions 231 and 252 in NahR (Huang and Schell 1991) or position 234 of OxyR (Christman et al., 1989) caused an inducer-independent phenotype, resembling the increase in light intensity in the transition between strains G2a and G3a, where Q233R was added (Figure 2C and Figure 9). In contrast, substitutions at NahR’s positions 227 or 253 resulted in activation-deficient mutants (Schell et al., 1990). These two different phenotypes emphasize the benefit of the random mutagenesis approach, given the high number of possible substitutions and the complexity in the *a priori* prediction of their effects.

From examination of the data in Figure 2 and Figure 3, it appears that the main improvement in detection sensitivity occurred during the first two rounds of random mutagenesis, from G0 to G2, while the main enhancement of signal intensity was contributed by mutations in the promoter region, as well as by changes in the reporter element and plasmid design. It could be postulated that changes to the coding sequence resulted in increased binding of the YhaJ transcription factor to its ligand, therefore increasing its sensitivity. Alternatively, changes to the promoter region resulted in increased production of YhaJ, leading to an overall increase in response but not necessarily to an increased detection sensitivity.

In an approach different from the one presented here, successful performance enhancement of bacterial reporters, involving a ligand-binding transcriptional regulator, has previously been reported by molecular redesign of the sensor’s response circuit, rather than by directed evolution. Small molecule-inducible gene expression was amplified by tuning intracellular receptor densities and the use of transcriptional amplifiers (Wang et al., 2014; Wang et al., 2015; Wan et al., 2019). Reducing the background signal while maintaining maximum output levels was achieved using protease-based post-translational degradation, which was put under the regulation of the cloned receptor, along with the reporter gene (Wan et al., 2019). Clearly, future optimal improvement of whole-cell sensor design should combine both approaches: a targeted redesign of specific circuit components interfaced with random modifications yielding beneficial effects that are difficult to predict.

Over 20 years ago, Burlage et al. (1999) were the first to propose the identification of the location of buried explosives by bacterial bioreporters engineered to respond by an optical signal to explosives’ vapors. For such a scheme to materialize, advances need to be made in two closely intertwined research directions–the development of the bacterial sensors on the one hand, and the engineering/optics involved in their imaging on the other hand. The present communication comprises another step towards the realization of the former objective. When contemplating such a field application for landmine detection employing luminescent bacterial sensor strains, a key factor would be the ability to perform rapid imaging, thus enabling the real-time scanning of large areas for the presence of buried explosives. A consequence of this need is for the bioreporters to produce high light intensities. In the final round of random mutagenesis, we have therefore focused on variants with a stronger luminescence, represented here by G3b, rather than on those exhibiting improved response ratios; the latter were characterized by low light emission under both induced and the non-induced conditions. Another significant factor that necessitates the field use of highly luminescent bioreporters is potential interference by ambient light. In a previous study (Shemer et al., 2021), imaging of the luminescent response of immobilized bioreporters spread on top of a buried antipersonnel landmine was possible only under complete darkness. One possible pathway towards a further reduction of the detection threshold involves the lowering of background luminescence; it is important that such an activity will also be accompanied with actions to increase signal intensity. An optimal balance between detection sensitivity and luminescence intensity will also depend upon the imaging apparatus employed, and its ability to separate the luminescent signal from ambient light.

## Data Availability

The original contributions presented in the study are included in the article/Supplementary Material; further inquiries can be directed to the corresponding author.

## Abbreviations

CDS, coding sequence; DNT, 2,4-dinitrotoluene; LTTR, LysR type transcription regulator; TNT, 2,4,6-trinitrotoluene.
