# Supplementary material for: Enhancing DNT Detection by a Bacterial Bioreporter: Directed Evolution of the Transcriptional Activator YhaJ
Source: Front Bioeng Biotechnol. 2022 Feb 14;10:821835. doi: 10.3389/fbioe.2022.821835 (PMC8882911; doi:10.3389/fbioe.2022.821835)
Supplement: Supplementary file 3 [file Table1.DOCX]

Table S 1: A full list of primers used in the course of this study

| Primer | Sequence (5’-3’) |
| --- | --- |
| yhaJ-SphI | TAATGCATGCACCATCCGTAGTCTGCTTGT |
| yhaJ-SalI | TAATGTCGACTTCTGGCAGCAATCGTTACG |
| 115_F | CTAACCAGTAAGTAATTACTTGAGAGGCCTTTCTGGCAGCAATCGTTACGGAAAC |
| 116_R | TTCGTTTTATTTGATGCCTGTCGACTACTAGAGCCTGCAGGACCATCCGTAGTCTGCTTGT |
| 117_yhaJ_RM | ACTACTAGAGCCTGCAGG |
| 118_yhaJ_RM | ATTACTTGAGAGGCCTTTCTGG |
